# Unraveling Riboflavin-Mediated Mitochondrial Modulation as a Therapeutic Pathway in Neurological Disorders: An Integrative Systematic Review

**DOI:** 10.1016/j.tjnut.2026.101427

**Published:** 2026-02-18

**Authors:** Eulália Rebeca Silva-Araújo, Ana Elisa Toscano, Henrique José Cavalcanti Bezerra Gouveia, Paula Brielle Pontes, Joaci Pereira dos Santos-Júnior, Osmar Henrique dos Santos-Júnior, Maria Daniele Teixeira Beltrão de Lemos, Nathalia Caroline de Oliveira Melo, Janaina Viana de Melo, Eduardo Padrón-Hernández, Raul Manhães-de-Castro

**Affiliations:** 1 Graduate Program in Neuropsychiatry and Behavioral Sciences, Center for Medical Sciences, Federal University of Pernambuco, Recife-Pernambuco, Brazil; 2 Studies in Nutrition and Phenotypic Plasticity Unit, Center for Health Sciences, Federal University of Pernambuco, Recife-Pernambuco, Brazil; 3 Laboratory of Mesomagnetics, Center for Exact and Natural Sciences, Federal University of Pernambuco, Recife-Pernambuco, Brazil; 4 Graduate Program in Nutrition, Center for Health Sciences, Federal University of Pernambuco, Recife-Pernambuco, Brazil; 5 Nursing Unit, Vitória Academic Center, Federal University of Pernambuco, Vitória de Santo Antão-Pernambuco, Brazil; 6 Department of Anatomy, Université du Québec à Trois-Rivières, Trois-Rivières, QC G8Z 4M3, Canada; 7 ESUDA University, Recife-Pernambuco, Brazil; 8 Department of Physics, Center for Exact and Natural Sciences, Federal University of Pernambuco, Recife-Pernambuco, Brazil

**Keywords:** mitochondria, vitamin B2, degenerative diseases, brain, mitochondrial biogenesis

## Abstract

Mitochondrial dysfunction is recognized as a key pathophysiological mechanism in neurodegenerative diseases. Alterations in mitochondrial dynamics—including imbalances in fission and fusion, impaired biogenesis, and disrupted mitophagy—contribute to the onset and progression of neurological disorders. In this context, mitochondrial modulation has emerged as a promising therapeutic strategy. This systematic review examined the role of riboflavin, a water-soluble vitamin and essential mitochondrial cofactor, in neurological interventions through mitochondrial modulation, with emphasis on elucidating the underlying molecular mechanisms. A search of the PubMed, Embase, Scopus, and Web of Science databases identified 23 eligible studies, comprising 6 in vitro experiments, 10 rodent models, and 7 clinical trials. These studies evaluated the effects of riboflavin in monogenic, neurodegenerative, and demyelinating mitochondrial diseases, cerebrovascular/hypoxic injury, and pain/migraine. Clinical evidence indicated that riboflavin may regulate oxidative stress in stroke and perinatal asphyxia, with associated functional improvements. Preclinical findings revealed mechanisms of action involving energy homeostasis, cell cycle regulation, and mitochondrial dynamics across monogenic mitochondrial disorders, neurodegenerative diseases, hypoxic injury, and models of pain and migraine. Possibly through mitochondrial modulation, riboflavin appeared to reduce α-synuclein aggregation in Parkinson’s disease, increase the number of tyrosine-hydroxylase-positive neurons in Alzheimer’s disease models, enhance neuronal survival in Brown-Vialetto–Van Laere and Huntington’s disease models, and normalize neuronal excitability in ataxia and migraine. In contrast, no therapeutic effects were observed in demyelinating diseases. Overall, the findings suggest that riboflavin may promote neuroprotection through redox modulation and gene regulation, stabilization of membrane potential, and enhanced mitochondrial complex activity via flavin cofactors, ultimately supporting neuronal metabolism and functional outcomes. Despite advances in mechanistic understanding, clinical applications in humans remain insufficiently defined for most conditions, with clearer dosage regimens currently established only for stroke and migraine.

## Introduction

In the central nervous system (CNS), proper biological functioning depends on energy homeostasis and maintenance of mitochondrial dynamics [[1], [2], [3]]. Dysregulation of these parameters, managed by mitochondria, can contribute to the development or exacerbation of neurological disorders, such as neurodegenerative diseases, migraines, strokes, and ataxia [[4], [5], [6], [7], [8]]. Mitochondrial dysfunction represents any alteration in the homeostatic functioning of mitochondria [9,10]. Several studies have proposed a link between mitochondrial dysfunction and neurological disorders [1,3,4,11]. In particular, neurodegeneration is associated with mitochondrial oxidative damage and energy failure, often resulting from the accumulation of misfolded proteins in the CNS [[11], [12], [13]].

Energy production is a mitochondrial function that is widely compromised in adult-onset neurological diseases, often caused by oxygen deprivation such as stroke [14]. This leads to oxidative stress, defined as an imbalance between the production of reactive oxygen species (ROS) and the organism’s antioxidant capacity (AOC) to neutralize them [15]. Furthermore, mitochondrial dysfunction alters membrane potential and neuronal excitability, with disruption of intracellular calcium homeostasis potentially exacerbating symptoms in epilepsy and Friedreich’s ataxia [4,14,16]. Changes in the cell cycle, including reduced mitochondrial biogenesis and increased mitophagy, are also observed in neurological conditions, notably Parkinson’s and Alzheimer’s diseases [17,18]. These impairments arise from dysregulation of genes controlling the cell cycle and mitochondrial dynamics during neurodegenerative processes [[19], [20], [21], [22], [23]]. Additionally, in aging-related diseases, mitochondrial dysfunction is particularly evident in T cells, triggering premature senescence, cell death, and cognitive decline [7,24,25].

Riboflavin is a water-soluble vitamin with antioxidant, anti-inflammatory, and neuroprotective properties [[26], [27], [28], [29], [30]]. In its cofactorial states, FAD and FMN, riboflavin participates in multiple reactions related to energy metabolism, including β-oxidation and oxidative phosphorylation [31]. It supports energy homeostasis and regulates the respiratory coefficient during ATP synthesis [32]. Additionally, riboflavin stimulates the reduction of energetic macromolecules within mitochondria, such as ATP and phosphocreatine, contributing to decreased oxidative stress [32]. Through interactions with the glutathione system and antioxidant enzymes like catalase (CAT) and superoxide dismutase (SOD), riboflavin helps regulate redox balance and intracellular calcium homeostasis [6,33,34]. Therefore, combining riboflavin with other B vitamins has been suggested as an alternative treatment for mitochondrial remodeling [35]. Riboflavin intervention promotes mitochondrial function by regulating oxidative stress and stimulating energy synthesis, aiding recovery from motor impairments seen in conditions such as ataxia and multiple sclerosis [14,36].

Clinical studies associated riboflavin supplementation with reductions in serum markers of mitochondrial oxidative stress and improvements in sensory, motor, and cognitive impairments characteristic of stroke [37,38]. Riboflavin treatment has promoted recovery from neurological symptoms, including motor coordination, while enhancing mitochondrial function in animal models of Alzheimer’s disease [39], and neuropathy [40]. Thus, riboflavin is implicated in restoring redox balance [6], energy homeostasis [41], cell cycle maintenance [5], and regulation of mitochondrial permeability [42], in neurological conditions. Previous systematic and narrative reviews have explored riboflavin’s therapeutic effects in stroke, multiple sclerosis, migraines, and Parkinson’s disease [26,43]. Both associate improvement in neurological outcomes with the antioxidant and neuroprotective roles of riboflavin at pharmacological doses [26,43]. Research into potential neuroprotective agents remains an active focus in neurodegenerative conditions [44,45]. Riboflavin’s role in myelin production—a lipid layer essential for saltatory synaptic conduction—has also been described as part of its mechanism of action [30,43].

Despite advances in understanding riboflavin’s effects on the nervous system, no systematic reviews have presented mitochondrial modulation as a therapeutic mechanism in neurological disorders. Mitochondrial modulation encompasses mechanisms that restore balance in mitochondrial dynamics, energy homeostasis, and oxidative stress [45]. This review synthesizes in vitro studies, animal models, and clinical trials to examine the effects of various riboflavin treatments on mitochondrial function—covering oxidative stress, energy homeostasis, cell cycle regulation, and mitochondrial dynamics—and their implications for symptomatic processes in neurological disorders.

## Methods

### Systematic review reporting guidelines and protocol registration

This systematic review includes in vitro studies, rodent models, and clinical trials. The manuscript follows the guidelines proposed by the PRISMA. The PROSPERO approved the protocols with registration numbers CRD42022361316 and CRD42022361672.

### Search strategy

A systematic literature search was conducted between March 2024 and June 2025 across the following databases: PubMed/Medline, Embase, Scopus, and Web of Science. Two independent reviewers (E.R.S.A. and O.H.S.J) performed the search, with a third reviewer (H.J.C.B.G.) resolving any disagreements. The search strategy was based on descriptors related to “intervention” and “outcomes,” as presented in Table 1. Primary outcomes included markers of oxidative stress, energy homeostasis, mitochondrial dynamics, and cell cycle. These comprised ROS levels, lipid peroxidation, antioxidant activity, energy production coefficient, mitochondrial complex activity, mitochondrial membrane potential, intracellular calcium levels, and/or activation of related receptors, as well as expression of genes associated with mitochondrial biogenesis or mitophagy. Secondary outcomes encompassed any parameters or manifestations related to the neurological disorder under study, including cognitive, motor, and sensory changes, as well as pathological mechanisms evaluated in vitro.

**TABLE 1:** Search terms

Parameter	Descriptors
Intervention	(riboflavin) OR (vitamin B-2) OR (flavin mononucleotide)
Outcomes	(mitochondria) OR (mitochondrial biogenesis) OR (mitophagy) OR (mitochondrial dynamics) OR (cellular respiration) OR (ATP synthesis) AND (brain) OR (cerebral) OR (nervous system) OR (cerebral disorders) OR (brain disorders) OR (degenerative) OR (neurodegenerative) OR (neurological disorder) OR (migraine)

This table presents the search terms used in the database to select studies.

### Eligibility and data extraction

The studies were selected through a 2-stage process: *1*) screening of titles and abstracts, and *2*) full-text review. Two independent reviewers (E.R.S.A. and O.H.S.J.) evaluated the studies based on eligibility criteria using the Population-Intervention-Comparison-Outcome-Study framework, presented in Table 2. Articles were imported and managed using a reference manager. After automatic removal of duplicates and inaccessible files, studies underwent the first screening phase and were excluded based on the following criteria: *1)* not an original article; *2)* not involving riboflavin; *3)* not related to neurological research; *4)* not an intervention study; *5)* population not of interest. In the second stage, studies passing the initial screening were reviewed in full and further excluded if they met any of the following: *6)* no riboflavin treatment; *7)* not focused on neurological disorders; *8)* no assessment of mitochondrial parameters. Finally, studies fulfilling all inclusion criteria were selected. From the included studies, the following data were extracted to create a “study characteristics” table: reference (author and year), study type (in vitro, animal model, or clinical trial), disease or condition (neurological disorder), population details (species, sex, age, groups), treatment specifics (substance, dose, duration, route of administration), and evaluated mitochondrial parameters. These mitochondrial parameters included oxidative stress markers; energy homeostasis indicators (energy production/use, membrane potential, ionic homeostasis); mitochondrial dynamics markers (shape, fusion, fission); and cell cycle parameters [mitochondrial DNA (mtDNA), biogenesis, mitophagy]. Extracted results were summarized in a separate table, categorized into “primary outcomes” (mitochondrial parameters) and/or “secondary outcomes” (neurological disorder-related parameters). The selection and extraction steps were conducted by 2 reviewers (E.R.S.A. and O.H.S.J.), with a third reviewer resolving any disagreements (H.J.C.B.G.).

**TABLE 2:** Eligibility criteria

PICOS strategy	Inclusion	Exclusion
Population	Rodents (rats and mice) or humans exposed to neurological disorders	Other rodents
Intervention	Riboflavin, FAD, or FMN (alone or in combination)	Other uncommon forms of riboflavin
Comparison	Control, placebo, or sham	No control group
Outcomes	Mitochondrial parameters, including oxidative stress, bioenergetic, mitochondrial dynamics, and cell cycle	Parameters are not associated with mitochondrial dysfunction in neurological diseases
Study	Preclinical model or clinical trial (in vivo or in vitro)	In silico and case reports

This table describes the inclusion and exclusion criteria applied in the selection of studies for this review according to the PICOS strategy.

Abbreviations: FAD, flavin adenine dinucleotide; FMN, flavin mononucleotide; PICOS, Population-Intervention-Comparison-Outcomes-Study.

### Risk of bias and study quality assessment

The adapted Quality Assessment Tool for In Vitro Studies tool was used to assess the risk of bias (RoB) in in vitro studies [46]. Studies with animal models were analyzed using the Systematic Review Center for Laboratory Animal Experimentation RoB tool [47]. The Cochrane RoB 2.0 tool was used for the methodological analysis of clinical trials [48]. These tools are categorized into 9 domains, encompassing selection bias, performance bias, detection bias, attrition bias, reporting bias, and other types of bias. The evaluation classified each domain as having “high risk,” “unclear risk,” or “low risk” of bias.

## Results

### Study selection

**FIGURE 1:**
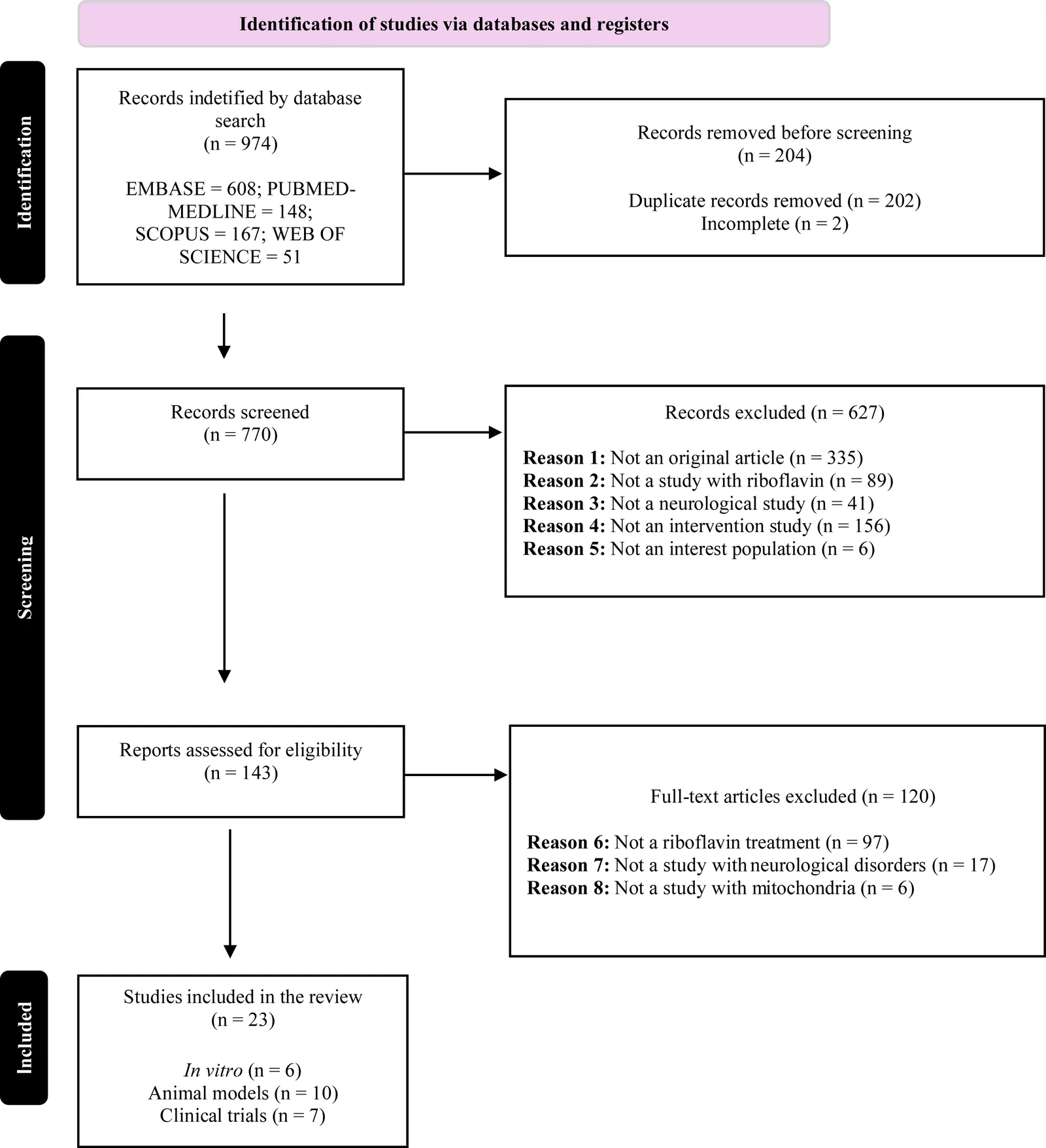
Diagram of the article selection process in this review. Stages: (1) identification, (2) screening, and (3) included.

In this review, a total of 974 studies were identified across databases: 148 in PubMed, 608 in Embase, 167 in Scopus, and 51 in Web of Science. After importing the records into a reference manager, 202 duplicates and 2 incomplete studies were automatically removed, leaving 770 articles for screening. Two independent reviewers then evaluated the studies in 2 stages. During the first stage, based on title and abstract screening, 627 studies were excluded according to the following criteria: *1)* not an original article (*n =* 335); *2)* not involving riboflavin (*n =* 89); *3)* not related to neurological research (*n =* 41); *4)* not an intervention study (*n =* 156); *5)* population not of interest (*n =* 6). Subsequently, 143 articles proceeded to the second screening stage, which involved full-text review. At this stage, 120 studies were excluded based on the following criteria: *6)* no riboflavin treatment (*n =* 97); *7)* not focused on neurological disorders (*n =* 17); *8)* no mitochondrial assessment (*n =* 6). Ultimately, 23 original articles met all inclusion criteria, comprising 6 in vitro studies, 10 rodent model studies, and 7 clinical trials in humans (Figure 1).

### Characteristics of in vitro studies

As shown in TABLE 3, TABLE 6 experimental in vitro studies were included in this review [16,[49], [50], [51], [52], [53]]. Among them, 3 studies modeled Brown’s syndrome [[49], [50], [51], 2[49–51], 2 reproduced Parkinson’s disease [52,53], and 1 used a Friedreich’s ataxia model [16]. All in vitro Brown’s syndrome models employed human pluripotent stem cell cultures treated with riboflavin [[49], [50], [51]]. To model Parkinson’s disease, 1 study used mouse midbrain dopaminergic neuronal cells line exposed to riboflavin treatment (via FMN cofactor) or control conditions [52]. Another study utilized Sloan–Kettering-Neuroblastoma-Metastatic Clavicle neuroblastoma cells treated with either a placebo or riboflavin at 3 different concentrations combined with other B vitamins [53]. The Friedreich’s ataxia model employed *S. cerevisiae* and *C. elegans* strains treated with various concentrations of riboflavin or its cofactors FMN and FAD [16]. Regarding mitochondrial parameters, cell cycle aspects were evaluated in 2 studies [49,53], bioenergetics in 2 studies [16,53], mitochondrial dynamics in 3 studies [[49], [50], [51]], and oxidative stress in 4 studies [[50], [51], [52], [53]] (Table 3).

**TABLE 3:** Characteristics of in vitro studies

Reference	Condition (disease)	Population (cells and groups)	Treatment and dosage
Colasuonno et al. (2020) [49]	Brown’s syndrome	Induced pluripotent stem cells derived from human fibroblasts	Riboflavin (1 μM)
Niceforo et al. (2021) [50]	Brown’s syndrome	Induced pluripotent stem cells	Riboflavin (10 μM)
Marioli et al. (2020) [51]	Brown’s syndrome	Patient-specific induced pluripotent stem cells	Riboflavin (75 mg/kg, in vivo treatment with the patient)
Gong et al. (2021) [52]	Parkinson’s disease	MN9D cells, subdivided into groups: Group 1: control Group 2: disease Group 3: disease + FMN	FMN (2.5 nM)
Gonzalez-Cabo et al. (2010) [16]	Friedreich’s ataxia	Strains of *Saccharomyces cerevisiae* and *Caenorhabditis elegans*	Riboflavin (0.01 mM, 0.1 mM, 0.5 mM, 1 mM), FMN (0.1 mM, 0.5 mM, 1 mM, 5 mM, 10 mM), or FAD (0.1 mM, 0.5 mM, 1 mM, 5 mM, 10 mM)
Jia et al. (2010) [53]	Parkinson’s disease	SK-N-MC neuroblastoma cells Group 1: control Group 2: treatment (1×) Group 3: treatment (2.5×) Group 4: treatment (5.0×) Group 5: treatment (10×)	Riboflavin (0.1, 0.35, 0.60 or 1.1 mg/L) + B1, B3, B6, B5 and B11

This table summarizes the main characteristics of the in vitro studies analyzed, including disease, sample type, and riboflavin treatment.

Abbreviations: FAD, flavine adenine dinucleotide; FMN, flavine mononucleotide; MN9D cells, mouse midbrain dopaminergic neuron cell; SK-N-MC, Sloan–Kettering-neuroblastoma-metastatic clavicle.

**TABLE 6:** Preclinical outcomes

Reference	Type of study and condition	Mitochondrial parameter evaluated	Mitochondrial outcomes	Secondary outcomes (signs/symptoms)
Colasuonno et al. (2020) [49]	In vitro Brown’s syndrome	Mitochondrial dynamics (shape) Cell cycle (mitochondrial biogenesis)	↓ Ultrastructural deformations in the mitochondria (swollen aspects and ruptured ridges) ↑ Number of mitochondria	No reported
Niceforo et al. (2021) [50]	In vitro Brown’s syndrome	Energy homeostasis (mitochondrial membrane potential)	↑ Cellular calcium influx	No reported
Marioli et al. (2020) [51]	In vitro Brown’s syndrome	Energy homeostasis (mitochondrial membrane potential)	↑ Cellular calcium influx (restores calcium homeostasis) ↓ Mitochondrial superoxide anions (restore cellular redox status)	No reported
Gong et al. (2021) [52]	In vitro Parkinson’s disease	Oxidative stress	FMN treatment: ↓ ROS ↓ GSSG ↑ SOD ↑ GSH	↑ TH+ neurons in the SNpc
Gonzalez-Cabo et al. (2010) [16]	In vitro Friedreich’s ataxia	Energy homeostasis	↑ Mitochondrial activity in complexes I, III, and IV by treatment with FAD ↑ ATP production (FAD and FMN)	↑ Muscle contraction (coordinated, cyclic activity, regulated by motor neurons)
Jia et al. (2010) [53]	In vitro Parkinson’s disease	Cell cycle (biogenesis mitochondrial, DNA protection) Energy homeostasis (complex activity) Oxidative stress	↑ PGC-1-α expression ↓ Oxidative damage to DNA ↑ Mitochondrial membrane potential (2.5× and 5×) ↑ Mitochondrial complex activity (2.5× and 5×) ↓ Carbonyl proteins (2.5× and 5×) ↓ ROS	↓ α-Synuclein and poly-ubiquitin
Peraza et al. (2018) [54]	Rodent model of Huntington’s disease	Oxidative stress	↓ GSH	↑ Cerebral dopamine levels
Moges et al. (2009) [55]	Rodent model of familial amyotrophic lateral sclerosis	Cellular damage due to oxidative stress	Unchanged cellular survival	Unchanged motor functions
Naghashpour et al. (2016) [36]	Rodent model of autoimmune encephalomyelitis	Mitochondrial dynamics (shape, fusion, fission)	↑ BDNF in the spinal cord and brain	↓ Motor disability in the chronic and effective phases of the disease
Liu et al. (2019) [56]	Rodent model of Alzheimer’s disease	Mitochondrial dynamics and cell cycle Oxidative stress	↓ 4-HNE, a marker of mitochondrial damage, including dysregulation in bioenergetics, calcium homeostasis, and mitochondrial dynamics ↓ 8-OHdG, a biomarker of mtDNA oxidative damage ↓ Nitrotyrosine (a marker of oxidative stress in mitochondrial membranes)	↑ Motor coordination ↑ Cognition
Visñuk et al. (2022) [39]	Rodent model of Alzheimer’s disease	Oxidative stress	↓ H_2_O_2_ (an endogenous ROS, produced in the mitochondria) (treatment with commercial riboflavin or CRL 2130 strain)	↑ TH+ cells in the SNpc (all treatments) ↑ Recovery of motor functions (all treatments)
Nazıroğlu et al. (2015) [42]	Rodent model of migraine	Energy homeostasis Oxidative stress	↑ Microsomal membrane activity Ca^(2+)^-ATPase (MMCA) ↓ Lipid peroxidation ↑ GSH ↑ GSH-Px	Preserved the wavelength on the electroencephalogram, with no abnormalities of spikes or discharges in the recordings
Stepanova et al. (2019) [41]	Rodent model of neonatal stroke	Energy homeostasis	↑ Mitochondrial complex I (NADH) activity	↓ Neurological deficiency
Bütün et al. (2014) [6]	Rodent model of migraine	Energy homeostasis Oxidative stress	↑ Brain microsomal Ca^2-^ATPase activity ↓ Lipid peroxidation ↑ GSH	No reported
Braga et al. (2020) [40]	Rodent model of neuropathy	Energy homeostasis	↑ The activity of potassium-dependent mitochondrial channels	↑ Motor coordination
Zhao et al. (2018) [5]	Rodent model of Alzheimer’s disease	Oxidative stress Cell cycle	Dose dependent: ↓ ROS ↑ GSH-Px activity ↑ Catalase activity ↑ Superoxide dismutase activity ↑ NRF-2 expression ↓ Keap1 expression (nullifies NRF-2 transcriptional activity and stimulates cell death)	↑ Memory and learning

This table summarizes the main outcomes evaluated and the main findings related to mitochondrial modulation in in vitro studies and rodent models.

Abbreviations: 4-HNE, 4-hydroxynonenal; 8-OHdG, 8-hydroxy-2'-deoxyguanosine; BDNF, brain-derived neurotrophic factor; CRL 2130, culture collection of the reference laboratory 2130; GSH, reduced glutathione; GSH-Px, glutathione peroxidases; GSSG, oxidized glutathione; H_2_O_2_, hydrogen peroxide; Keap1, Kelch-like ECH-associated protein 1; MDA, malondialdehyde; MMCA, microsomal membrane activity Ca(2+)-ATPase; mtDNA, mitochondrial DNA; NRF2, nuclear factor erythroid 2-related factor-2; PGC-1α, peroxisome proliferator-activated receptor γ coactivator 1-α; ROS, reactive oxygen species; SNpc, substantia nigra; SOD, superoxide dismutase; TH+, tyrosine hydroxylase in positive.

### Characteristics of animal model studies

This review included 10 preclinical studies conducted in rodents (Table 4) [5,6,[39], [40], [41], [42],^50^,[54], [55], [56], [57]]. Three of these studies simulated Alzheimer’s disease [5,39,56], and 2 established migraine models [6,42]. Single studies modeled Huntington’s disease [54], familial amyotrophic lateral sclerosis [55], experimental autoimmune encephalomyelitis [57], stroke [41], and neuropathy [40]. The Alzheimer’s studies used female and male amyloid precursor protein 23 mice [56], male C57 Black Laboratory, strain 6 (C57BL/6) mice [39], and male amyloid precursor protein/presenilin 1 mice [5]. C57BL/6 mice were also used in the experimental autoimmune encephalomyelitis [57], and the stroke model [41]. Glycine-to-alanine substitution at codon 93/SOD 1 transgenic mice were employed for the familial amyotrophic lateral sclerosis model [55], whereas male Swiss mice were used in the neuropathy model [40]. Female Wistar rats were used in the 2 migraine studies [6,42].

**TABLE 4:** Characteristics of rodent model studies

Reference	Condition (disease)	Population (species and groups)	Treatment (dosage, duration, and route)
Peraza et al. (2018) [54]	Huntington’s disease	Male Fisher rats were grouped (*n* = 6) Group 1: healthy control Group 2: disease Group 3: riboflavin Group 4: disease + riboflavin Group 5–6: placebo	Riboflavin (10 mg/kg) Duration: 5 d Intraperitoneally route
Moges et al. (2009) [55]	Familial amyotrophic lateral sclerosis	G93A/SOD1 transgenic mice (*n* = 45) Group 1: control Group 2 and 4: riboflavin Group 3: placebo	Riboflavin (12 mg/kg, in the diet) Duration: 51 d to death Oral route
Naghashpour et al. (2016) [36]	Experimental autoimmune encephalomyelitis	C57BL/6 female mice (*n* = 56) Group 1 and 2: sham Group 3 and 4: disease + placebo Group 5: disease + interferon β-1a Group 6: disease + riboflavin Group 7: disease + interferon β-1a + riboflavin	Riboflavin (10 mg/kg) Duration: 2 wk Oral gavage
Liu et al. (2019) [56]	Alzheimer’s disease	APP23 mice (*n* = 23 males and 22 females) Groups 1 and 2: healthy controls Group 3: disease + placebo Group 4: disease + treatment	Twendee X (riboflavin + other compounds) Duration: 4.5–12 mo Oral gavage
Visñuk et al. (2022) [39]	Alzheimer’s disease	Male C57BL/6 mice, age: 8 wk (*n* = 35) Group 1: control Group 2: placebo Group 3: disease + riboflavin Group 4: disease + riboflavin-producing strain (*L. plantarum* CRL 2130) Group 5: disease + riboflavin-producing strain (*L. plantarum* CRL 725)	Riboflavin (1.6 μg/mL) *L. plantarum* CRL 2130 (295 ± 30 ng/mL) *L. plantarum* CRL 725 (91 ± 12 ng/mL) Duration: 5 d Oral route
Zhao et al. (2018) [5]	Alzheimer’s disease	Male APP/PS1 double transgenic mice, age: 3 mo (*n* = 60) Group 1 and 2: control Group 3: placebo Group 4: disease+ riboflavin (10 mg/kg) Group 5: disease + riboflavin (30 mg/kg)	Riboflavin (10 or 30 mg/kg) Duration: 70 d Intragastric route
Nazıroğlu et al. (2015) [42]	Migraine	Female Wistar rats (*n* = 32) Group 1: control Group 2: disease Group 3: disease + riboflavin + selenium Group 4: disease + selenium	Riboflavin (100 mg/kg) + selenium (1.5 mg/kg) Duration: 10 d Oral route
Bütün et al. (2014) [6]	Migraine	Female Wistar rats, 8–12 wk (*n* = 60) Group 1: control Group 2: disease Group 3: disease + riboflavin Group 4: disease + riboflavin + vitamin E	Riboflavin (100 mg/kg) Duration: 10 d before migraine Oral route
Stepanova et al. (2019) [41]	Neonatal stroke	C57BL/6 mice, 7 d (*n* = 7–12/group) Group 1: placebo Group 2: disease + riboflavin	Riboflavin (25 mg/kg) Duration: 5 injections containing 5 mg/kg, at 24, 12 and 1.5 h before the insult and at 0 and 1 h after the insult Intraperitoneal route
Braga et al. (2020) [40]	Neuropathy	Male Swiss mice (*n* = 24) Group 1: placebo Group 2: disease + riboflavin Group 3: disease + thiamine Group 4: disease + nicotinamide	Riboflavin (500 mg/kg) Duration: 2 applications every 2 h after 7 d of insult Oral route

This table summarizes the main characteristics of the in vivo rodent studies, including disease, sample type, and riboflavin treatment.

Abbreviations: APP23, amyloid precursor protein 23; APP/PS1, amyloid precursor protein/presenilin 1; CRL, culture collection of the reference laboratory; C57BL/6, C57 Black Laboratory, substrain 6; G93A/SOD1, glycine-to-alanine substitution at codon 93/superoxide dismutase 1.

Except for 1 study with only 2 experimental groups [41], all articles included negative and positive control groups alongside groups receiving different riboflavin treatments. In 8 studies, riboflavin was administered alone, with treatment durations ranging from 5 to 70 d [5,6,39,41,42,54,55,57]. Among these, 5 studies also analyzed groups receiving treatments other than riboflavin separately [6,40,54,55,57] (Table 4).

Riboflavin was administered in combination with other compounds in only 2 studies: via the drug TwendeeX [56], or in combination with a low dose of selenium [42]. Administration routes included gavage in 2 studies [50,57], oral route in 5 studies [6,39,40,42,55], intraperitoneally in 2 studies [41,54], and intragastrically in 1 study [5] (Table 4).

### Characteristics of clinical trials

As shown in TABLE 5, TABLE 7 clinical trials were included in this review [33,37,38,[58], [59], [60], [61]]. Multiple sclerosis and perinatal asphyxia were each investigated in 1 trial [57,59], with the study populations consisting of young adults and neonates, respectively. The remaining 5 studies involved elderly patients with stroke [33,38,60,61]. In all clinical trials, participants were assigned to either a riboflavin or a placebo group. Riboflavin was administered at a dose of 5 mg in all studies, except for the multiple sclerosis trial, which used a 10 mg dose [57]. Treatment duration ranged from 10 to 14 d [33,38,[59], [60], [61]] to 6 mo in the case of the multiple sclerosis study [57]. Administration routes included oral and/or intravenous delivery. Oxidative stress was the only mitochondrial parameter assessed across all trials (Table 5).

**TABLE 5:** Characteristics of clinical trials

Reference	Condition (disease)	Population (sample, age, and groups)	Treatment (dosage, duration, and route)
Naghashpour et al. (2013) [58]	Multiple sclerosis	29 patients Age: 31–33 y old Group 1: placebo Group 2: disease + treatment	Riboflavin (10 mg) Duration: 6 mo Oral route
Skoromets et al. (2016) [59]	Perinatal asphyxia	89 patients Age: 0.7–30.0 d Group 1: control Group 2: disease + treatment	Riboflavin (5 mg), through the drug cytoflavin Duration: 10 d Intravenous route
Rumiantseva et al. (2010) [60]	Hemorrhagic stroke	30 patients Age: 44–75.0 y old Group 1: control (*n* = 14) Group 2: disease + treatment (*n* = 16)	Riboflavin (5 mg), through the drug cytoflavin Duration: 35 d Intravenous and oral routes
Silina et al. (2021) [61]	Hemorrhagic stroke	151 patients Age: 47–74.3 y old Group 1: control (*n =* 79) Group 2: disease + treatment (*n* = 72)	Riboflavin (5 mg), through the drug cytoflavin Duration: 10 d Intravenous route
Ullegaddi et al. (2006) [38]	Stroke	48 patients (*n* = 24 per group) 30 males and 28 females Age: 63–84 y old Group 1: placebo Group 2: disease + treatment	Riboflavin (5 mg) + B-6, B-9 and B-12 vitamins Duration: 14 d Oral route
Ullegaddi et al. (2004) [37]	Stroke	48 patients (*n* = 24 per group) Age: not informed Group 1: placebo Group 2: disease + treatment	Riboflavin (5 mg) + B-6, B-9 and B-12 vitamins Duration: 14 d Oral route
Gariballa and Ullegaddi (2007) [33]	Stroke	97 patients, both sexes Age: 67–84 y old Group 1: placebo Group 2: disease + treatment	Riboflavin (5 mg) + B-6, B-9 and B-12 vitamins Duration: 14 d Oral route

This table summarizes the main characteristics of the clinical trials analyzed, including disease, population, and riboflavin treatment.

**TABLE 7:** Clinical outcomes

Reference	Type of study and condition	Mitochondrial parameter evaluated	Mitochondrial outcomes	Secondary outcomes (signs/symptoms)
Naghashpour et al. (2013) [58]	Clinical trial of multiple sclerosis	Oxidative stress	Unchanged EGRAC compared with the control patients	Unchanged functional status compared with the control group
Skoromets et al. (2016) [59]	Clinical trial of perinatal asphyxia	Oxidative stress	↓ Plasma lactate levels, a marker of mitochondrial oxidative stress	↑ Recuperation of head circumference ↑ Sensorimotor functions ↑ Regulation of reflex responses
Rumiantseva et al. (2010) [60]	Clinical trial of hemorrhagic stroke	Oxidative stress	↓ MDA, a marker of mitochondrial dysfunction, increases ROS and degrades proteins in mitochondria	↓ Progression of cerebral infarction ↓ Alteration of the cerebral hemispheres Recuperation of cognitive and functional status (Glasgow Scale and Barthel Index)
Silina et al. (2021) [61]	Clinical trial of hemorrhagic stroke	Oxidative stress	↓ MDA	↓ Neurological deficiency ↓ Hematoma brain
Gariballa and Ullegaddi (2007) [33]	Clinical trial of stroke	Oxidative stress and energy homeostasis	↑ Antioxidant function	No results
Ullegaddi et al. (2006) [38]	Clinical trial of stroke	Oxidative stress	↓ MDA	↑ Cognitive and functional capacity
Ullegaddi et al. (2004) [37]	Clinical trial of stroke	Oxidative stress	↓ MDA ↑ TAOC	↑ Functional independence (Barthel scale)

This table summarizes the main outcomes evaluated and the main findings related to mitochondrial modulation in clinical trials.

Abbreviations: EGRAC, erythrocyte glutathione reductase activity coefficient; MDA, malondialdehyde; ROS, reactive oxygen species; TAOC, total antioxidant capacity.

### Main findings

#### Monogenic mitochondrial disorders

As shown in Table 6, riboflavin treatment reduced mitochondrial superoxide anion levels and restored cellular redox status in an in vitro model of Brown’s syndrome [51] (Tables 3 and 6). In an in vitro model of Friedrich’s ataxia, treatment with FAD, a riboflavin cofactor, stimulated the activity of mitochondrial complexes I, III, and IV, designated respectively as NADH, cytochrome bc1, and cytochrome-c oxidase [16] (Tables 3 and 6). Also in this study, riboflavin in its 2 cofactor states (FAD and FMN) increased ATP synthesis without altering the oxidative balance [16] (Table 6). In addition, 2 in vitro studies reported that riboflavin treatment stimulated increased cellular calcium influx in Brown’s syndrome [50,51] (Tables 3 and 6). Riboflavin reduced mitochondrial ultrastructural deformations and promoted mitochondrial biogenesis in cultured in vitro cells derived from patients with Brown’s syndrome [49] (Tables 3 and 6). Few studies were found on riboflavin treatment in monogenic mitochondrial diseases, and the evidence gathered, according to the scope of this review, was of a preclinical nature. This may indicate that the use of riboflavin in these conditions is still in its early stages or reflects the absence of biomolecular assessments in existing clinical trials, which led to their exclusion during the search process.

#### Neurodegenerative disorders

Treatment with FMN, a cofactor form of riboflavin, decreased ROS and oxidized glutathione in an in vitro model of Parkinson’s disease [52] (Tables 3 and 6). Moreover, it enhanced antioxidant activity by increasing the expression of SOD and reduced glutathione (GSH) [53]. In an animal model of Alzheimer’s disease, riboflavin effectively reduced ROS levels, specifically hydrogen peroxide (H_2_O_2_), a byproduct of mitochondrial metabolism [39] (Tables 4 and 6).

Another study with an Alzheimer’s model showed that riboflavin reduced ROS and increased the activity of antioxidant enzymes SOD, CAT, and glutathione peroxidase (GSH-Px) in a dose-dependent manner [5] (Tables 4 and 6). In contrast, in a Huntington’s disease model, riboflavin supplementation enhanced brain energy synthesis but reduced GSH levels, thereby preventing free radical formation [54] (Table 6). The increased GSH observed in Parkinson’s disease and migraine may reflect an early compensatory antioxidant response to oxidative stress [6,52], whereas in Huntington’s disease [54], GSH depletion may result from severe mitochondrial dysfunction and compromised redox regulation. Taken together, these findings suggest that riboflavin therapy may exert differential effects depending on the underlying pathophysiological condition.

In the context of Parkinson’s disease, an in vitro study also reported an increase in mitochondrial complex activity following treatment with different concentrations of riboflavin [53] (Tables 3 and 6). Moreover, in an animal model of Alzheimer’s disease, riboflavin contributed to energy homeostasis by reducing the expression of 4-hydroxynonenal (4-HNE), a marker of mitochondrial damage associated with energy [56] (Table 6). In summary, these studies highlight the role of riboflavin in regulating mitochondrial complexes through its cofactors FAD and FMN. These cofactors participate in enzymatic reactions involved in the Krebs cycle, DNA synthesis, β-oxidation, and protein degradation [31].

As shown in Tables 3 and 6, treatment with different doses of riboflavin increased mitochondrial membrane potential in an in vitro model of Parkinson’s disease [53]. In this study, riboflavin treatment increased the gene expression of peroxisome proliferator-activated receptor γ coactivator 1-α, a key marker of mitochondrial biogenesis, and also reduced oxidative DNA damage [53] (Table 6). In animals subjected to an Alzheimer’s disease model, calcium homeostasis was restored following treatment with a medication containing riboflavin in its composition [56] (Tables 4 and 6). Riboflavin also decreased the expression of 8-hydroxy-2'-deoxyguanosine, a biomarker of mtDNA oxidation [56] (Tables 4 and 6).

Additionally, high-dose riboflavin increased the expression of nuclear factor erythroid 2-related factor 2, a transcription factor that regulates mitochondrial function, and reduced cell death by inactivating kelch-like ECH-associated protein 1, a mediator of protein ubiquitination, in an animal model of Alzheimer’s disease [5] (Tables 4 and 6). Although these are preclinical studies, these findings provide important mechanistic information about the effects of riboflavin therapy in the context of degenerative diseases. However, the scarcity of clinical evidence limits our understanding of key aspects, such as dose and frequency, regarding the modulation of mitochondrial function by riboflavin in the neurodegenerative context in humans.

#### Vascular/hypoxic brain injury

In clinical trials with stroke patients, combined administration of riboflavin and other B-complex vitamins increased total AOC and reduced serum malondialdehyde (MDA), a biomarker of mitochondrial dysfunction associated with elevated ROS [33,37,38] (Tables 5 and 7). Supporting the mechanistic understanding of riboflavin’s antioxidant action, FMN treatment was shown to stimulate mitochondrial complex I activity in an animal model of stroke [41] (Table 6). In human neonates affected by perinatal asphyxia, riboflavin treatment reduced serum lactate levels, another marker of oxidative stress and metabolic acidosis [59] (Tables 5 and 7). However, evidence in this condition is limited to a single study, highlighting the need for future clinical trials during critical developmental windows.

#### Demyelinating diseases

In this review, a preclinical study using a small animal sample found no changes in oxidative stress following riboflavin treatment in amyotrophic lateral sclerosis [55] (Tables 4 and 6). Additionally, a clinical trial found no association between riboflavin supplementation and improved antioxidant function, as erythrocyte glutathione reductase activity coefficient remained unchanged in patients with multiple sclerosis [58] (Tables 5 and 7). Another included evidences suggest that riboflavin stimulated the expression of brain-derived neurotrophic factor (BDNF), a gene associated with mitochondrial biogenesis, in the brain and spinal cord of rodents with experimental autoimmune encephalomyelitis [36] (Tables 4 and 6). Conversely, neuronal damage induced by oxidative stress in a familial amyotrophic lateral sclerosis model remained unaltered by riboflavin treatment alone, showing improvement only when combined with light therapy [55]. The gathered evidence appears to suggest that riboflavin has limited effects in demyelinating diseases, which may be explained by mitochondrial mechanisms being a secondary process in these conditions.

#### Pain or migraine

In an animal model of neuropathy, riboflavin administration effectively regulated the activity of potassium-dependent mitochondrial channels and modulated cellular excitability [40] (Tables 4 and 6). Dysregulation of mitochondrial membrane potential impairs ATP synthesis, whereas alterations in ion channel function contribute to CNS oxidative damage and apoptosis [40]. Moreover, 2 studies using an animal model of migraine reported a decrease in lipid peroxidation and increased expression of antioxidant enzymes, including GSH and GSH-Px, following riboflavin treatment [6,42] (Tables 4 and 6).

In both studies, calcium homeostasis was achieved through increased activity of brain microsomal Ca^2+^-ATPase, which regulates membrane depolarization [6,42] (Table 6). Alterations in neuronal excitability and depolarization underlie the pathophysiology of neuropathies and migraines [6,40,42]. The gathered evidence clarifies the mechanistic actions of riboflavin in these conditions and may provide a basis for future clinical research.

#### Pathophysiological recovery through mitochondrial modulation

Riboflavin administration increases tyrosine hydroxylase (TH+) expression in neurons of the substantia nigra pars compacta (SNpc) in an in vitro model of Parkinson’s disease [52] (Tables 3 and 6), contributing to tyrosine metabolism and dopaminergic transmission. In response to riboflavin, dopamine levels in the brain were also accentuated following increased bioenergetics and reduced oxidative stress in Huntington’s disease [54] (Table 6). Riboflavin administration also promoted stress regulation, bioenergetic homeostasis, and cell cycle maintenance, in addition to reducing α-synuclein and polyubiquitin levels in an in vitro model of Parkinson’s disease [53] (Tables 3 and 6). Moreover, the regulation of oxidative stress and mitochondrial dynamics induced by riboflavin treatment increased TH+ cell counts in the SNpc and contributed to motor function recovery in rodents subjected to Alzheimer’s disease [39] (Tables 4 and 6). In another study, enhanced bioenergetics stimulated muscle contraction and improved performance in coordinated and cyclical motor activities regulated by motor neurons, as shown in an in vitro model of Friedreich’s ataxia using *S. cerevisiae* and *C. elegans* strains [16] (Tables 3 and 6). In the context of neurodegeneration, improvements in memory, learning, motor performance, and/or cognition were reported alongside the regulation of mitochondrial function and cell cycle dynamics in models of Friedreich’s ataxia [16], and Parkinson’s [53], and Alzheimer’s disease [5,56] (Tables 3, 4, and 6).

In humans, supplementation with 5 mg of riboflavin improved AOC and supported patients’ cognitive and functional recovery after stroke [37,38,61] (Tables 5 and 7). In cases of perinatal asphyxia, riboflavin was associated with reduced oxidative stress, sensorimotor recovery, and normalization of head circumference [59] (Tables 5 and 7). Riboflavin also promoted ontogenesis and maturation of sensorimotor reflexes in rodents subjected to stroke [41] (Tables 4 and 6), strengthening the clinical findings gathered in vascular and hypoxic brain injuries.

Mitochondrial modulation by riboflavin may have preserved electrical activity in the electroencephalogram, which showed no spike or discharge abnormalities in rodents exposed to an experimental migraine model [42] (Tables 4 and 6). In a neuropathy model, mitochondrial dynamics regulation combined with restored bioenergetic homeostasis was associated with improved motor performance in mice treated with riboflavin-containing formulations [40] (Tables 4 and 6). Overall, the included studies reported improvements in mitochondrial function associated with the recovery of signs and symptoms characteristic of monogenic and neurodegenerative mitochondrial diseases, vascular or hypoxic damage, and pain/migraine evaluated in this review, while evidence in demyelinating diseases remained limited. However, heterogeneity within and between disease subtypes should be taken into account when extrapolating these findings to human populations.

#### Overview of riboflavin treatment parameters

In this review, the conditions examined were largely diverse with respect to treatment characteristics (dose, posology, and route of administration), as well as study population or experimental model, which limits broader comparisons regarding the most effective dosing strategies. On the basis of the available preclinical evidence, only a limited inference can be drawn, as merely 2 independent studies investigated the same dose—100 mg/kg of riboflavin administered orally for 10 consecutive days—in a rodent model of migraine to assess mitochondrial function [6,42]. The remaining preclinical conditions varied substantially in dose, treatment frequency, and experimental design (Tables 4 and 6).

When compared with clinical studies—although these were not included in the main body of the review due to eligibility criteria—it becomes evident that a commonly used dosage and frequency for migraine prophylaxis in adults is relatively well established, with documented tolerability and efficacy [62,63]. Additionally, among the clinical trials included in this review, a dose of 5 mg of riboflavin combined with other compounds was reported as part of a treatment strategy for stroke; however, the timing of administration ranged from 10 to 35 d postinjury [33,37,38,60,61], preventing more definitive conclusions about dosing patterns in clinical practice. In contrast, only 1 eligible study involving multiple sclerosis, using a 10 mg dose, met the inclusion criteria [58], limiting the extent to which dose and frequency can be discussed for this condition (Tables 5 and 7).

### RoB

**FIGURE 2:**
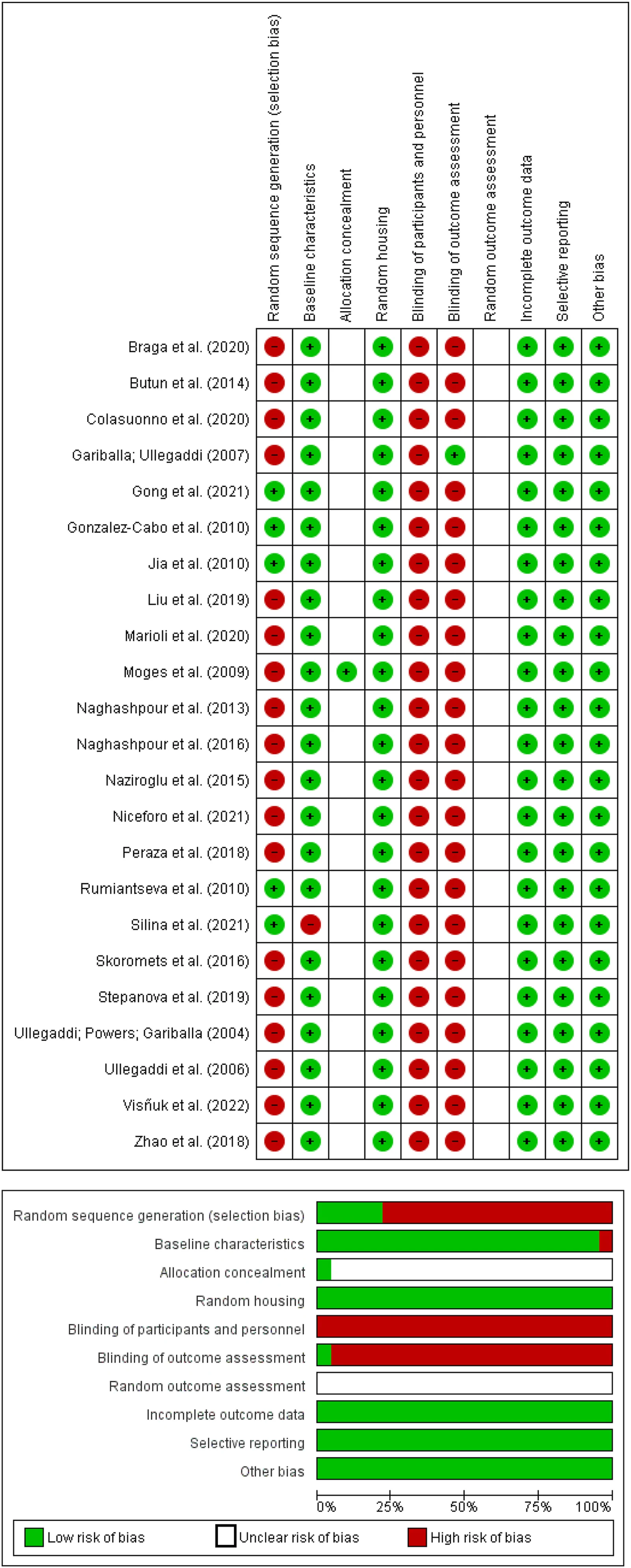
Risk of bias summary. Risk of bias assessment of in vitro studies using QUIN’s tool (adapted), of rodent models using SYRCLE’s tool, and of clinical studies using the Cochrane RoB 2.0 tool. The studies are presented in alphabetical order and were organized according to the Review Manager software. QUIN, Quality Assessment Tool for in Vitro Studies; SYRCLE’s, Systematic Review Center for Laboratory Animal Experimentation.

Methodological analysis in the studies is presented in Figure 2. In this review, the RoB in random sequence generation was low for 50% in vitro studies. For baseline characteristics, all studies presented a low RoB (100%). None of the evaluated studies provided clear information on allocation concealment. Furthermore, all studies had a low risk of random allocation bias (100%). On the other hand, the most prevalent bias corresponded to the blinding of participants and staff, which is one of the domains of selection bias. In this domain, all studies presented a high RoB. Despite this, it is worth highlighting that blindness is less common in studies with animal models, which represent the majority of the work included in this review. The blinding bias of the results was low for only 1 clinical trial, representing 7.7% of the total studies. No study provided accurate information for the detection domain, named random outcome assessment (0.0%). However, all articles showed a low-risk bias for the following 3 domains (100%): random placement, incomplete outcome data, and selective outcome reporting.

## Discussion

This review integrates preclinical and clinical studies investigating the effects of riboflavin as a mitochondrial modulator across a broad spectrum of neurological disorders. The included evidence encompasses monogenic mitochondrial diseases (e.g., Brown–Vialetto syndrome, Friedreich’s ataxia); neurodegenerative proteinopathies (Parkinson’s, Alzheimer’s, and Huntington’s diseases); vascular and hypoxic brain injuries (stroke and perinatal asphyxia); demyelinating diseases (amyotrophic lateral sclerosis and multiple sclerosis); and pain/migraine conditions.

Across these studies, riboflavin interventions appear to attenuate oxidative stress in humans with vascular or hypoxic injuries, while promoting energy homeostasis and modulating mitochondrial dynamics in preclinical models of several neurological subtypes. These mitochondrial actions have been linked to improvements in pathophysiological processes, including enhanced neuronal survival in monogenic mitochondrial disorders; normalization of neuronal action potentials in pain/migraine; reduced protein aggregation in the CNS and regulation of dopaminergic pathways in neurodegenerative diseases; and recovery of cognitive and sensorimotor functions reported in both preclinical and clinical studies of monogenic mitochondrial diseases, neurodegenerative disorders, pain/migraine, and vascular/hypoxic injury. In contrast, the effects of riboflavin in demyelinating diseases appear limited or absent. Available studies have not demonstrated meaningful benefits in slowing disease progression, restoring function, or modifying pathophysiological markers [55,58]. This may reflect the secondary role of mitochondrial dysfunction in the pathophysiology of demyelinating disorders [64], in which targeting mitochondrial pathways alone is unlikely to yield clinically relevant improvements. Overall, although preclinical studies have been instrumental in elucidating mechanistic pathways, translating these findings to human clinical practice requires caution. Clinical evidence remains scarce and diversified, underscoring the need for future studies investigating optimal dosing, treatment duration, and efficacy across the neurological conditions explored.

In this review, riboflavin treatment appears to attenuate oxidative stress in neurodegenerative disorders through its interaction with H_2_O_2_ levels, as demonstrated in preclinical studies of Alzheimer’s disease [39]. Altered H_2_O_2_ homeostasis has been previously linked to impaired energy regulation in Alzheimer’s, affecting both ATP production and utilization while promoting a redox imbalance that exacerbates cognitive decline, neuronal aging, and oxidative damage [65]. Although our systematic review offers mechanistic insights into the potential role of riboflavin in Alzheimer’s disease, clinical evidence remains scarce, underscoring the need for future studies to validate and extend these preclinical observations.

Reductions in oxidative stress have also been reported in preclinical models of Parkinson’s and Huntington’s diseases following riboflavin treatment [52,54]. Lowering ROS levels in degenerative diseases may influence both energetic and epigenetic pathways, potentially mitigating cognitive and sensorimotor decline associated with neuronal aging [66]. However, behavioral parameters were not assessed in the studies reviewed, limiting the extent of interpretation regarding functional outcomes.

Expanding the understanding of mitochondrial modulation in neurodegenerative disorders, riboflavin treatment enhanced mitochondrial complex activity in a rodent model of Parkinson’s disease, contributing to pathophysiological improvement [53]. Stabilizing Parkinson’s disease requires sustained dopaminergic activity, which imposes substantial metabolic stress on mitochondria to maintain energy production and essential cellular processes such as autophagy, fusion, and fission [67].

Extending these mechanistic insights in neurodegenerative diseases, preclinical evidence from rodent models suggests that riboflavin treatment, by activating genes associated with mitochondrial function, can inhibit mtDNA oxidation in an Alzheimer’s disease model [56], and enhance synaptic plasticity through upregulation of BDNF, in Huntington’s disease [54]. The PGC-1-α/BDNF pathway is an established therapeutic target in the previous literature for neuroprotection and mitochondrial modulation in neurodegenerative disease [68]. Given the preclinical nature of these findings, future clinical studies are encouraged to correlate the expression of these mitochondrial biogenesis markers in peripheral blood cells with behavioral outcomes and riboflavin’s pharmacological efficacy, aiming to clarify the translational relevance of these pathways in human neurological conditions.

Mitochondrial support provided by riboflavin may also help restore redox balance in Alzheimer’s disease, mitigating lipid peroxidation and cellular damage through reductions in 4-HNE levels in rodent models [56]. Elevated 4-HNE, together with MDA and NADPHoxidase-4, has been shown to impair mitochondrial ATP generation in the cerebral cortex, as demonstrated in genetically modified Alzheimer’s models [69]. Overall, these preclinical findings offer mechanistic explanations suggesting that riboflavin modulates mitochondrial function across neurodegenerative diseases, with possible implications for slowing disease progression.

In pain and migraine conditions, oxidative stress has been proposed as a therapeutic target, particularly in cases involving aura episodes—characterized by perceptual disturbances such as visual phenomena [70]. Migraines may arise from an imbalance between cerebral energy demand and supply, and recovery appears to depend on restoring energy homeostasis and reducing oxidative stress [71]. In this review, 2 preclinical migraine models demonstrated reductions in ROS and increases in antioxidant enzyme activity, possibly in response to riboflavin treatment [6,42]. These mechanistic findings align with the pathophysiology of migraine, in which stabilizing neuronal membrane potential and regulating pain transmission require substantial energy expenditure by the cortical sodium/potassium ATPase pump, one of the brain’s highest energy consumers [6,42]. Although this review included only studies focused on mitochondrial modulation due to our predefined inclusion criteria, the clinical use of riboflavin for migraine prophylaxis in humans is well established in the literature, typically at a dose of 400 mg/d for 3–6 mo [62,63,72]. Future reviews addressing these studies are timely and will support the findings gathered in this work.

A high cerebral energy demand is also observed in vascular or hypoxic brain injuries, both during and after periods of oxygen deprivation [41]. Therefore, therapies that support redox homeostasis are beneficial and may aid postinjury recovery. In stroke, the clinical trials included in this review reported biochemical indications of increased AOC and reduced oxidative and metabolic stress after riboflavin treatment [33,37,38]. Riboflavin also appeared to contribute to redox rebalancing, attenuation of lactic acidosis, and modulation of inflammatory responses triggered by injury in children with perinatal asphyxia in a clinical trial [26]. Extending the mechanistic understanding of riboflavin in this subtype of neurological damage, preclinical evidence demonstrated that riboflavin may support neurogenic processes, potentially contributing to functional recovery in conditions associated with cerebral oxygen deprivation [26,32]. Among the clinical trials conducted under hemorrhagic or hypoxic conditions, improvements in cognitive function were reported in 4 stroke studies associated with redox homeostasis [37,38,60,61], whereas only 1 study found no behavioral changes [58]. The antioxidant action of riboflavin may also explain the normalization of head circumference and sensorimotor function observed in cases of perinatal asphyxia reported in 1 included clinical trial [59].

Riboflavin degrades into FAD and FMN in the body, through which it participates in the assembly of mitochondrial complexes and acts as a cofactor in multiple enzymatic reactions [43]. Elevated levels of FAD/FMN in the mitochondrial matrix are known to be associated with increased energy production and utilization, as well as with the regulation of calcium influx [73]. In an in vitro ataxia model, treatment with FAD or FMN appears to increase proton-pumping and electron transport chain activity, thereby enhancing mitochondrial complex function [16]. These processes increase ATP synthesis and modulate oxidative stress during energy expenditure, contributing to a mechanism known as energy homeostasis [74]. Considering these mechanisms, preclinical evidence suggests that restoring energy homeostasis through treatment with riboflavin-derived cofactors promotes the recovery of sensorimotor reflexes and increases neuronal survival [16]. FMN/FAD helps reduce oxidative stress by modulating the glutathione system through activation of glutathione reductase, which in turn inhibits peroxide reactions [26]. Additional in vitro evidence indicates that riboflavin-mediated modulation of oxidative stress may involve increased SOD expression, contributing to the maintenance of neuronal mitochondrial function in Brown’s syndrome [49]. Overall, in monogenic mitochondrial diseases, riboflavin appears to exert antioxidant actions that support ionic homeostasis—an essential requirement for maintaining mitochondrial coupling and redox balance.

Another in vitro findings show that FMN may reduce oxidative stress markers in Parkinson’s disease model, including ROS levels [53], as it improves the protective functions of the CNS through the activation of antioxidant enzymes, such as SOD and CAT, which help modulate the oxidative balance [5,34]. The accumulation of α-synuclein in Parkinson’s disease—a hallmark of the disorder [49]—disrupts mitochondrial membrane protein receptors, specifically the translocases of the outer membrane (Tom20 and Tom22), leading to mitochondrial dysfunction, energy deficits, oxidative stress, and loss of mitochondrial membrane potential [75]. In this review, riboflavin appeared to reduce α-synuclein expression by modulating mitochondrial membrane potential in an in vitro model of Parkinson’s disease [49]. This effect occurs because riboflavin, in its FAD and FMN forms, participates in the respiratory chain, maintaining the proton-motive force and ensuring the stability of membrane potential-dependent channels [76]. Possibly through this mechanism, riboflavin may also influence ion homeostasis in monogenic mitochondrial disorders, as observed in in vitro studies of Brown–Vialetto syndrome [50,51], in degenerative diseases such as Alzheimer’s disease model [56], and in pain/migraine conditions [6,40,42].

Although these mechanisms have been elucidated in preclinical studies, their clinical relevance remains to be established and should be investigated in human studies. Because direct assessment of ion channel activity and membrane depolarization in vivo is challenging, future studies may utilize indirect approaches—such as electroencephalography, evoked potentials, and functional magnetic resonance imaging—to evaluate riboflavin dosage and efficacy under these conditions.

In our review, all included studies addressed the use of riboflavin in a pharmacological context, with doses exceeding dietary recommendations. No studies on supplementation for correcting nutritional deficiencies were found in the search or included within the scope of this review. Daily riboflavin requirements range from 0.3 to 1.4 mg/d depending on life stage, and upper intake limits have not been established, suggesting that riboflavin is tolerable even at high doses [77]. At basal levels, riboflavin supports metabolic and hematological functions, as well as vision and tissue formation [78]. At doses above the recommended dietary allowance, riboflavin has demonstrated effects of neuromodulation and, although classified as a vitamin, it can be employed in specific contexts to support pharmacotherapeutic interventions. Future studies should evaluate nutritional status before and after treatment to investigate potential associations between riboflavin metabolism and specific neurological conditions. This is an important limitation of the included studies, which, while highlighting pharmacological use due to high doses, may overlook preexisting deficiencies. This review suggests that baseline nutritional status and the relationship between habitual riboflavin intake, plasma biomarkers, and therapeutic response should be considered in the clinical context.

Riboflavin is primarily metabolized in the urinary system [78,79]. However, depending on the route of administration, the body may excrete it through products other than urine, including feces [80]. It should be noted that half-life varies according to the route of administration [79,80], but studies on riboflavin metabolism are still inconclusive. It is understood that riboflavin, even in high doses, is nontoxic [77,78], and its use as a pharmacological agent has been demonstrated in animals at doses ≤100 mg/kg [27,81], and in humans, ≤1200 mg/d [82]. Although extrapolating to humans requires caution, preclinical studies provide essential insights into dose optimization, safety, and tolerability, particularly in disease-specific contexts where clinical data are still limited. Although little has been explored regarding safe and effective high-dose regimens in humans, the dose commonly used for migraine prophylaxis has been investigated in previous studies [62,63,72]. Continuous treatment with 400 mg of riboflavin for 3 and 6 mo reduced headache frequency from 4 d/mo at baseline to 2 d/mo after both 3 and 6 mo in adults [62]. This dose was examined in another clinical trial, which demonstrated that 3 mo of treatment significantly reduced the frequency of migraine attacks and the number of headache days in adults [63]. Despite the limited solubility of riboflavin at high doses, its use as a prophylactic strategy for headaches—at doses considered high—has been described as safe, tolerable, and effective in adolescents and adults, according to existing clinical trials and a previous systematic review [72]. In addition, our review suggests that a 5 mg dose of riboflavin, when administered in combination with other compounds—although not in isolation—shows promise in treating stroke in humans [37,38,60,61]. Nevertheless, the treatment frequency reported across studies was varied, underscoring the need for further research to clarify optimal dosing frequency. The findings presented suggest that riboflavin is a promising intervention; however, further clarification is required regarding its use in humans, particularly in underexplored clinical contexts. Establishing an optimal dosage for the conditions addressed in this review—Alzheimer’s, Parkinson’s, Brown–Vialetto syndrome, ataxias, and various forms of sclerosis—remains a significant gap that warrants attention in future investigations. Robust clinical studies are needed to more precisely define appropriate dosage regimens, routes of administration, and therapeutic efficacy. Moreover, the use of riboflavin-producing lactic acid bacteria alongside conventional treatment has recently been highlighted in the literature as a strategy that may enhance the therapeutic efficacy of riboflavin [83]. This approach should be considered in future interventions for neurological conditions.

In conclusion, this review provides the molecular basis for the potential actions of riboflavin in neurological disorders, encompassing the early mechanistic stages largely reflected in preclinical research. Although our work highlights the current scarcity of clinical investigations involving riboflavin across the neurological conditions examined, it also identifies promising avenues that may be explored in future, condition-specific clinical applications.

## Author contributions

The authors’ responsibilities were as follows – ERS-A: designed research, conducted research, analyzed data, and wrote paper; AET: designed research, provided essential materials, had primary responsibility for final content; HJCBG: designed research; PBP: analyzed data and wrote paper; JPdSJ: provided essential materials and analyzed data; OHdS-J: provided essential materials and analyzed data; MDTBdL: analyzed data; NCdOM: analyzed data; JVdM: analyzed data and had primary responsibility for final content; EP-H: designed research, provided essential materials, and had primary responsibility for final content; RM-d-C: designed research, provided essential materials, and had primary responsibility for final content; and all authors: read and approved the final manuscript.

## Data availability

Data described in the manuscript, codebook, and analytic code will be made available on request pending application and approval.

## Funding

This study was funded in part by the “Fundação de Amparo à Ciência e Tecnologia de Pernambuco” (FACEPE, #0989-4.05/22 and #1471-4.05/22)—Brazil, “Conselho Nacional de Desenvolvimento Científico e Tecnológico” (CNPq) (#404181/2023-6)—Brazil, and “Coordenação de Aperfeiçoamento de Pessoal de Nível Superior”—Brazil (CAPES)—(Finance Code 001 and 1238/2022 /88881.707895/2022-01). These funding sources were not involved in the design, execution, analysis, and interpretation of the data in this study.

## Conflict of interest

The authors report no conflicts of interest.
